# Bortezomib, Mitoxantrone Hydrochloride Liposome, and Dexamethasone for Relapsed/Refractory Multiple Myeloma: A Multi‐Center, Open‐Label Phase I Trial

**DOI:** 10.1002/cam4.70890

**Published:** 2025-04-15

**Authors:** Ya‐Lan Zhou, Jin‐Qiao Zhang, Wei Wang, Li Bao, Bai‐Jun Fang, Da Gao, Li‐Ping Su, Wen‐Ming Chen, Guang‐Zhong Yang

**Affiliations:** ^1^ Department of Hematology, Beijing Chaoyang Hospital Capital Medical University Beijing China; ^2^ Department of Hematology The Third Hospital of Hebei Medical University Hebei China; ^3^ Department of Hematology The Second Affiliated Hospital of Harbin Medical University Heilongjiang China; ^4^ Department of Hematology, Beijing Jishuitan Hospital Capital Medical University Beijing China; ^5^ Department of Hematology Affiliated Cancer Hospital of Zhengzhou University and Henan Cancer Hospital Henan China; ^6^ Department of Hematology Affiliated Hospital of Inner Mongolia Medical University Inner Mongolia China; ^7^ Department of Hematology Shanxi Provincial Cancer Hospital Shanxi China

**Keywords:** efficacy, mitoxantrone hydrochloride liposome, relapsed/refractory multiple myeloma, safety

## Abstract

**Backgrounds:**

Mitoxantrone hydrochloride liposome (Lipo‐MIT) has shown clinical benefits in various tumors. However, there is no prospective study evaluating its effectiveness and safety in relapsed/refractory multiple myeloma (RRMM). A phase I trial of bortezomib, Lipo‐MIT, and dexamethasone (VMitD) with the primary endpoints being safety and efficacy was performed.

**Methods:**

Twenty subjects were enrolled this study, and the dose of Lipo‐MIT was designed to be 12, 16, and 20 mg/m^2^ at Day 1 combined with bortezomib and dexamethasone.

**Results:**

The most common grade 3/4 non‐hematologic adverse event was pneumonia (20%). The most frequently observed grade 3/4 hematologic toxicity included thrombocytopenia (70%), neutropenia (55%), lymphopenia (30%), and anemia (10%). Fifteen subjects received at least one efficacy evaluation, including 60% (9/15) with a very good partial response (VGPR) or better, resulting in an overall response rate (ORR) of 86.7% (13/15).

**Conclusions:**

This is the first report about the novel triplet regimen VMitD, including Lipo‐MIT for RRMM, which was well tolerated and demonstrated efficacy. Further studies are required to assess the outcomes more accurately and to evaluate its effectiveness in comparison to other salvage regimens containing proteasome inhibitors and anthracyclines.

**Trial Registration:** ClinicalTrials.gov identifier: NCT05052970

## 1 Introduction

Multiple myeloma (MM) is an incurable hematologic cancer characterized by the unregulated proliferation of malignant plasma cells, making it the second most prevalent hematological malignancy [1]. Over the past decade, significant progress has been made in the development of treatment options for relapsed or refractory multiple myeloma (RRMM). Despite these advancements, however, multiple myeloma remains an incurable disease [1, 2, 3].

As a cell cycle non‐specific anti‐tumor drug, mitoxantrone exerts its anti‐tumor effect by intercalating into deoxyribonucleic acid, leading to cross‐linking and fragmentation of the DNA structure, interfering with RNA, and inhibiting topoisomerase II [4]. Recent advancements in chemotherapy agents formulated with liposomes have enhanced anti‐tumor efficacy while reducing associated toxicity. Mitoxantrone hydrochloride liposome (Lipo‐MIT) has been approved in China for treating patients with relapsed or refractory peripheral T‐cell lymphoma. It has demonstrated strong antitumor effects in patients with the NKTCL subtype, overcoming the inherent resistance of NKTCL to anthracyclines [5]. Recently, Jiao et al. performed a clinical analysis of Lipo‐MIT in the treatment of pediatric patients with high‐risk acute myeloid leukemia, demonstrating comparable efficacy to idarubicin while offering improved safety [6]. However, as far as we know, there are few reports on the application of Lipo‐MIT in RRMM.

The first proteasome inhibitor of its kind, bortezomib, was approved for clinical use after a phase II trial demonstrated a 35% overall response rate (ORR) and a median progression‐free survival (PFS) of seven months in patients with RRMM treated with bortezomib monotherapy [7]. Since that time, various trials have assessed the use of bortezomib in combination with other therapeutic agents [8]. Preclinical and clinical data from various combination regimens suggest that bortezomib enhances the sensitivity of myeloma cells to other agents, leading to synergistic effects with various anti‐tumor drugs, including anthracyclines [9].

Considering the superior anti‐tumor efficacy of Lipo‐MIT compared to conventional anthracyclines, we hypothesized that combining bortezomib with Lipo‐MIT could be effective. To evaluate this, we undertook a prospective phase I clinical trial (NCT05052970) of the bortezomib, Lipo‐MIT, and dexamethasone (VMitD) regimen. The primary endpoints of the trial were to assess the safety and efficacy of the combination for RRMM.

## 2 Materials and Methods

A multi‐center, open‐label, phase I clinical trial was conducted to evaluate the combination of Lipo‐MIT, bortezomib, and dexamethasone (referred to as VMitD hereafter). Lipo‐MIT was given on day 1 of each 21‐day cycle. Bortezomib was administered on days 1, 4, 8, and 11, while dexamethasone was given on Days 1, 2, 4, 5, 8, 9, 11, and 12. Up to eight cycles of VMitD induction treatment were planned for administration. Subsequently, patients continued maintenance therapy with bortezomib twice weekly until disease progression or the onset of intolerable toxicity.

The study was approved by the Ethics Committee of Beijing Chaoyang Hospital, Capital Medical University, and written informed consent was obtained from all participants in accordance with the principles outlined in the Declaration of Helsinki.

Eligible patients were those aged 18 years or older with a diagnosis of RRMM who had received one or more prior treatments and with measurable disease. Exclusion criteria included compromised hematologic reserve, liver dysfunction, or diminished performance status, as well as the need for hemodialysis. Additionally, patients with a history of plasma cell leukemia, HIV, or active hepatitis were excluded from the study. Subjects who had received prior bortezomib but without resistance or intolerance were included. In addition, subjects who had previously received pegylated liposomal doxorubicin (PLD) or other anthracyclines were also eligible for inclusion.

**FIGURE 1:**
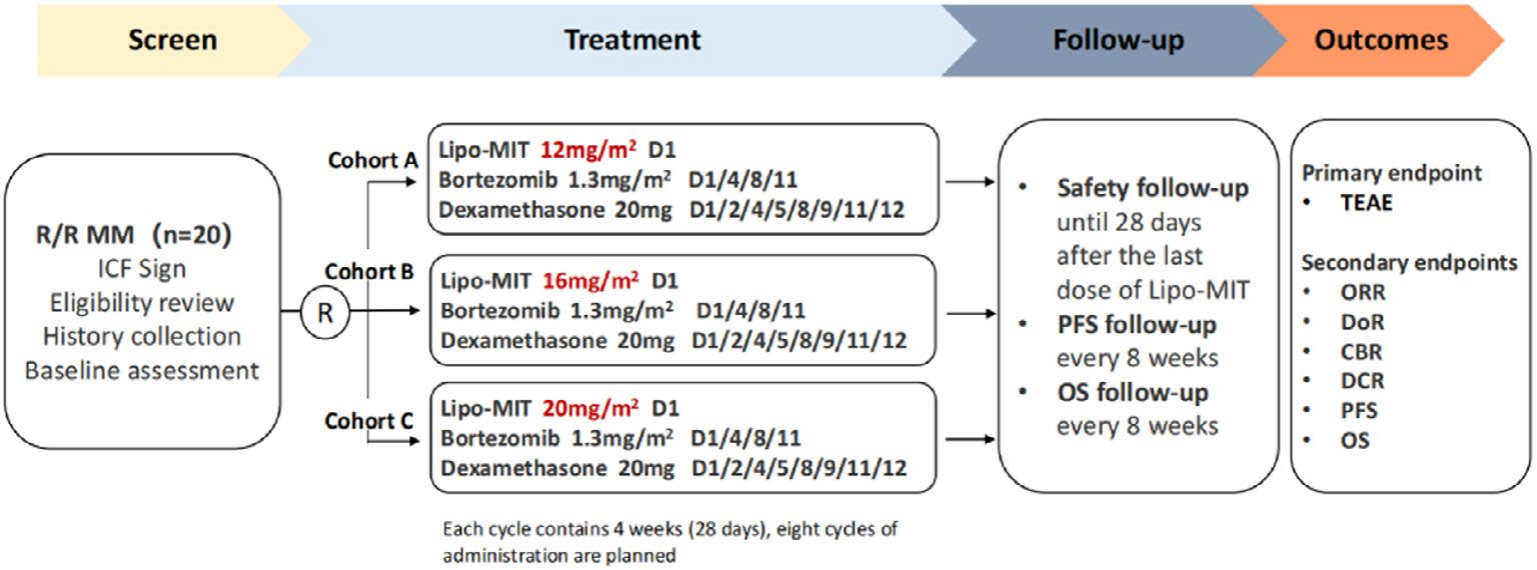
Study design flow chart.

Subjects were randomly assigned to three cohorts at a 1:1:1 ratio to receive different doses of Lipo‐MIT, with a total of 20 cases in three cohorts. The dosing regimen and schedule are outlined in Figure 1. The primary objective of this study was to assess the safety of combining Lipo‐MIT injection with bortezomib and dexamethasone for the treatment of RRMM.

## 3 Results

### 3.1 Baseline Characteristics of Subject‐ and Disease‐Related Covariates

As of March 2024, a total of 20 subjects participated in the study, and their baseline characteristics are presented in Table 1. The median age of the study cohort was 61.5 years, with a range of 42–70 years, and 40% of participants were female. 55% exhibited the IgG isotype, which was the most common isotype, followed by IgA isotype (30%), light chain isotype (10%), and others (5%). At the initial stage of the study, 80% of the subjects were Durie‐Salmon (DS) III, while 55% were further stratified as stage III by the revised‐international staging system (R‐ISS). Twenty percent of the cases involved MM with measurable extramedullary plasmacytoma (EMP). Among the 20 subjects, 90% had relapsed disease, and 10% had refractory disease before treatment. The median number of previous treatment lines was 2.5, ranging from 1 to 8, with 80% of patients having received bortezomib, 75% lenalidomide, 65% PLD or epirubicin, 60% cyclophosphamide, 30% thalidomide, 15% pomalidomide, 15% ixazomib, and 15% having undergone a prior autologous transplant. First‐line treatment regimens of the 20 patients are presented in the Supporting Information.

**TABLE 1:** Baseline characteristics of subject‐ and disease‐related co‐variates.

	Cohort A	Cohort B	Cohort C	Overall
	(*N* = 6)	(*N* = 7)	(*N* = 7)	(*N* = 20)
Age, years [median (range)]	62 (54, 66)	61 (49, 69)	55 (42, 70)	61.5 (42, 70)
Male, *n* (%)	3 (50.0)	4 (57.1)	5 (71.4)	12 (60.0)
Female, *n* (%)	3 (50.0)	3 (42.9)	2 (28.6)	8 (40.0)
Type of multiple myeloma, *n* (%)				
IgG	3 (50.0)	4 (57.1)	4 (57.1)	11 (55.0)
IgA	2 (33.3)	2 (28.6)	2 (28.6)	6 (30.0)
Serum free light‐chain only	0	1 (14.3)	1 (14.3)	2 (10.0)
Other	1 (16.7)	0	0	1 (5.0)
Durie‐Salmon disease stage, *n* (%)				
I‐A	1 (16.7)	0	0	1 (5.0)
II‐A	2 (33.3)	0	1 (14.3)	3 (15.0)
III‐A	3 (50.0)	6 (85.7)	4 (57.1)	13 (65.0)
III‐B	0	1 (14.3)	2 (28.6)	3 (15.0)
Revised‐International Staging System, *n* (%)				
I	3 (50.0)	1 (14.3)	1 (14.3)	5 (25.0)
II	0	1 (14.3)	2 (28.6)	3 (15.0)
III	3 (50.0)	4 (57.1)	4 (57.1)	11 (55.0)
Unknown	0	1 (14.3)	0	1 (5.0)
Extramedullary plasmacytoma, *n* (%)				
Yes	1 (16.7)	2 (28.6)	1 (14.3)	4 (20.0)
No	5 (83.3)	5 (71.4)	6 (85.7)	16 (80.0)
Eastern Cooperative Oncology Group performance status, *n* (%)				
0	1 (16.7)	3 (42.9)	3 (42.9)	7 (35.0)
1	2 (33.3)	2 (28.6)	4 (57.1)	8 (40.0)
2	3 (50.0)	2 (28.6)	0	5 (25.0)
High‐risk cytogenetic abnormality, *n* (%)				
Del17p	0	2 (28.6)	0	2 (10.0)
t(4;14)	0	3 (42.9)	0	3 (15.0)
Number of previous lines of therapy, median (range)	2.0 (1–4)	2.0 (2–4)	5.0 (1–8)	2.5 (1–8)
Disease category, *n* (%)				
Relapsed	4 (66.7)	7 (100)	7 (100)	18 (90.0)
Refractory	2 (33.3)	0	0	2 (10.0)

### 3.2 Efficacy Assessments

**FIGURE 2:**
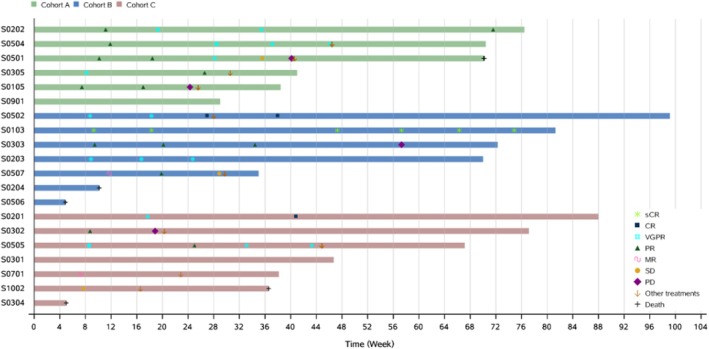
Swimmer plot of RRMM treated with VMitD.

**FIGURE 3:**
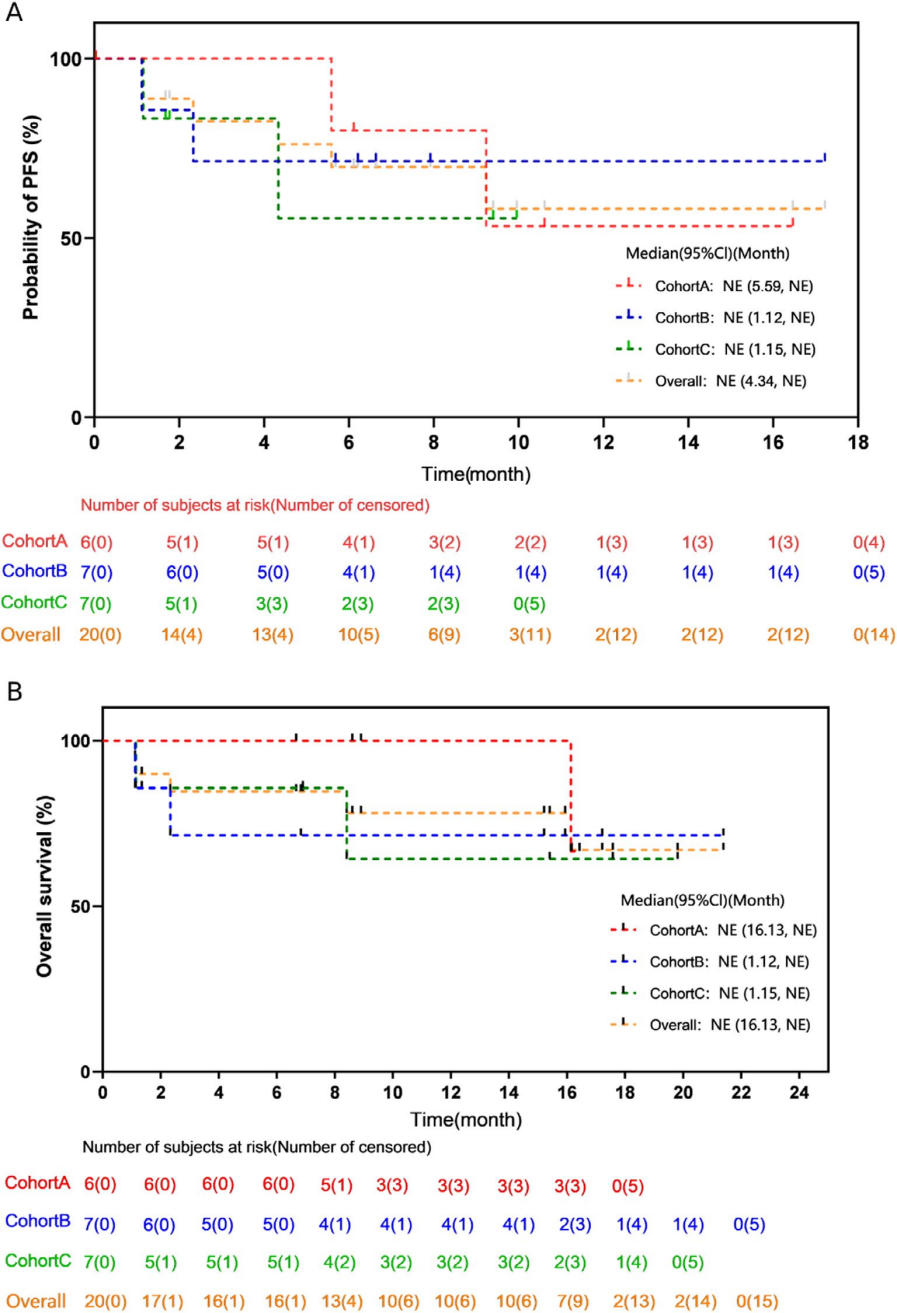
Progression‐free and overall survival curves (A) PFS plot; (B) OS plot.

The median number of cycles given was 6.3, with a range of 1.4 to 14.8. Overall, 15 subjects had received at least one efficacy evaluation, including three with a complete response (CR) or stringent CR (sCR), six very good partial response (VGPR), four partial remission (PR), one minor remission (MR) and 1 stable disease (SD), resulting in an ORR of 86.7% (13/15) in the efficacy evaluable analysis set as displayed in Figure 2. The duration of response (DoR) rate at 12 months was 66.0% (95% CI 23.92–88.63). The 12‐month PFS rate was 58.2% (95% CI 26.9–79.9). With a median follow‐up of 12.1 months, the median overall survival (OS) has not been achieved, and the estimated 12‐month OS rate stands at 78.2% (95% CI 51.36–91.32). The data on efficacy are presented in Table 2, with the survival plots shown in Figures 2 and 3. Of the four patients with extramedullary plasmacytoma, three underwent at least one efficacy evaluation, which included one case of sCR and two VGPR. One patient died before the first evaluation due to disease progression.

**TABLE 2:** Efficacy data of VMitD regimen in RRMM.

	Cohort A	Cohort B	Cohort C	Overall
	(*N* = 6)	(*N* = 7)	(*N* = 7)	(*N* = 20)
Best overall response (BOR), *n* (%)				
Stringent complete response	0	1 (14.3)	0	1 (5.0)
Complete response	0	1 (14.3)	1 (14.3)	2 (10.0)
Very good partial response	4 (66.7)	1 (14.3)	1 (14.3)	6 (30.0)
Partial response	1 (16.7)	2 (28.6)	1 (14.3)	4 (20.0)
Minimal response	0	0	1 (14.3)	1 (5.0)
Stable disease	0	0	1 (14.3)	1 (5.0)
Progressive disease	0	0	0	0
Response could not be evaluated	1 (16.7)	2 (28.6)	2 (28.6)	5 (25.0)
Duration of response rate in 12 months (%)	53.3 (6.83, 86.31)	100 (100.00, 100.00)	NE (NE, NE)	66.0 (23.92, 88.63)
PFS rate in 12 months (%)	53.3 (6.83, 86.31)	71.4 (25.82, 91.98)	NE (NE, NE)	58.2 (26.94, 79.98)
OS rate in 12 months (%)	100 (100.00, 100.00)	71.4 (25.82, 91.98)	64.3 (15.15, 90.17)	78.2 (51.36, 91.32)

### 3.3 Toxicity

Mild to moderate hematologic or non‐hematologic toxicities were frequently observed, whereas severe cases were rare. No treatment‐related adverse event (TRAE) related to research drugs leading to death occurred (Table 3). Infectious pneumonia (20%) was the only common grade 3/4 non‐hematologic toxicity observed. Grade 3/4 hematologic adverse events frequently observed included thrombocytopenia (70%), neutropenia (55%), lymphopenia (30%), and anemia (10%). Severe occurrences were rare and were typically managed with transfusions, leading to dose reductions in seven subjects (35%). Considering the potential cardiac risk of anthracyclines, treatment‐related cardiac toxicity was monitored during the treatment, which was all recorded in grade 1 or 2. Among the five patients without efficacy evaluation, three patients died before the first treatment evaluation due to one case of disease progression, one case of coronavirus infection disease, and one case of cardiac arrest, respectively. Two patients withdrew from treatment without treatment evaluation for personal reasons. All the above events were unrelated to the VMitD regimen.

**TABLE 3:** Treatment‐related adverse events grade ≥ 3 in the safety population.

Events	Cohort A	Cohort B	Cohort C	Overall
	(*N* = 6)	(*N* = 7)	(*N* = 7)	(*N* = 20)
Treatment‐related adverse events, *n* (%)	5 (83.3)	7 (100)	5 (71.4)	17 (85.0)
Thrombocytopenia, *n* (%)	4 (66.7)	5 (71.4)	5 (71.4)	14 (70.0)
Neutropenia, *n* (%)	3 (50.0)	5 (71.4)	3 (42.9)	11 (55.0)
Leucopenia, *n* (%)	3 (50.0)	5 (71.4)	2 (28.6)	10 (50.0)
Lymphopenia, *n* (%)	2 (33.3)	3 (42.9)	1 (14.3)	6 (30.0)
Infectious pneumonia, *n* (%)	3 (50.0)	0	1 (14.3)	4 (20.0)
Anemia, *n* (%)	0	0	2 (28.6)	2 (10.0)
Hypokalemia, *n* (%)	0	1 (14.3)	1 (14.3)	2 (10.0)
Hyponatremia, *n* (%)	0	0	1 (14.3)	1 (5.0)
Headache, *n* (%)	0	1 (14.3)	0	1 (5.0)
Diarrhea, *n* (%)	1 (16.7)	0	0	1 (5.0)

## 4 Discussion

In this study, we for the first time conducted a prospective phase I trial using a triplet regimen with Lipo‐MIT, bortezomib, and dexamethasone in RRMM patients, which showed good tolerance and efficacy. The estimated ORR is 86.7% with 60% (9/15) having a VGPR or better. The DoR rate in 12 months was 66.0% (95% CI 23.92–88.63). The 12‐month PFS rate was 58.2% (95% CI 26.9–79.9), while the 12‐month OS rate was 78.2% (95% CI 51.36–91.32), suggesting the three‐drug regimen as a promising salvage treatment choice for RRMM.

Although direct comparisons between trials are challenging, it is essential to interpret the results within the context of other three‐drug salvage regimens containing a proteasome inhibitor and anthracycline, like VDD (bortezomib, PLD, and dexamethasone) and KDD (carfilzomib, PLD, and dexamethasone). The ORR of 86.7% seen with VMitD is much better than that of the VDD regimen with an ORR of 44% in the randomized phase III study conducted by Orlowski et al. [10]. Similarly, Schroeder et al. performed a phase I/II trial of KDD [11], which resulted in an ORR rate of 83% in the cohort of patients treated at the maximum tolerated dose, suggesting a comparable efficacy with the VMitD study reported here. More importantly, VMitD also appeared to induce more profound responses, with a CR/sCR/VGPR rate of 60%. In addition, the efficacy of the VMitD regimen is also comparable when compared with novel therapies such as the Daratumumab combined regimen, with a range of ORR between 82.9% and 84% [12, 13]. It is worth noting that this regimen appeared to be highly effective in patients with RRMM and extramedullary lesions. Of the 20 patients, four presented with extramedullary lesions at enrollment, with two achieving sCR, one achieving VGPR following treatment, as well as 1 without efficacy evaluation because of disease progression.

The VMitD combination seemed to be well tolerated. No treatment‐related adverse event (TRAE) related to research drugs leading to death occurred. Thrombocytopenia (70%) was the most common grade 3/4 hematologic toxicity observed. This was higher than the incidence of thrombocytopenia (23%) in the combination of VDD [10]. In comparison, the combination of KDD resulted in similar rates of hematologic adverse events, including lymphopenia (63%), thrombocytopenia (40%), anemia (40%), and neutropenia (28%) [11].

This study has several limitations. Firstly, the early termination and small sample size restrict the ability to conduct further analyses, such as multivariate analysis. However, the preliminary data provide critical insights into dose‐dependent safety profiles and preliminary efficacy signals, which will inform the design of future trials. Secondly, subjects received diverse prior therapies, and assignment to transplant was not random. Thirdly, we only conducted basic clinical analysis and did not further explore the mechanism behind the regimen or investigate potential biomarkers that may predict response to this regimen. Despite the limitations, the prospective trial design endows this study with strong evidence strength and the data suggest that VMitD may represent a promising salvage therapy option for patients with RRMM beyond the second line.

In conclusion, this trial is the first to present outcomes using a triplet regimen including Lipo‐MIT for RRMM, demonstrating good tolerance and promising efficacy. However, further studies are required to more accurately assess patient outcomes, and in‐depth mechanistic studies are needed to identify potential biomarkers that could predict response to this regimen.

## Author Contributions

**Ya‐Lan Zhou:** writing – original draft, conceptualization, methodology, data curation, investigation and validation. **Jin‐Qiao Zhang:** conceptualization, writing – original draft, data curation and validation. **Wei Wang:** conceptualization, writing – original draft, data curation and validation. **Li Bao:** conceptualization, writing – original draft, data curation and validation. **Bai‐Jun Fang:** conceptualization, writing – original draft, data curation and validation. **Da Gao:** conceptualization, writing – original draft, data curation and validation. **Li‐Ping Su:** conceptualization, writing – original draft, data curation and validation. **Wen‐Ming Chen:** conceptualization, writing – original draft, data curation and validation. **Guang‐Zhong Yang:** conceptualization, writing – original draft, writing – review and editing, methodology, data curation, supervision, resources, project administration, funding acquisition, investigation and validation.

## Conflicts of Interest

The authors declare no conflicts of interest.

## Supporting information


Data S1.

## Data Availability

The datasets used in this study are available from the corresponding author on reasonable request.
