# Supplementary material for: Bortezomib, Mitoxantrone Hydrochloride Liposome, and Dexamethasone for Relapsed/Refractory Multiple Myeloma: A Multi‐Center, Open‐Label Phase I Trial
Source: Cancer Med. 2025 Apr 15;14(8):e70890. doi: 10.1002/cam4.70890 (PMC11998603; doi:10.1002/cam4.70890)
Supplement: Supplementary file 1 — Data S1. [file CAM4-14-e70890-s001.docx]

**Inclusion and exclusion criteria**

The inclusion criteria were:

1. Patients fully understand and voluntarily participate in this study and sign informed consent;
2. Aged 18-75 years, without gender limitation;
3. Patients with relapsed or refractory multiple myeloma (confirmed by histologically or cytologically) who had received at least one prior line regular treatment;
4. Patients have at least one of the following conditions: (1) Serum M protein≥10g/L; (2) Urine M protein≥200 mg/24h; (3) Serum free light chain(sFLC): κ/λ FLC ratio is abnormal and affected FLC ≥100mg /L;
5. Eastern Cooperative Oncology Group (ECOG) performance status of 0 to 2;
6. Laboratory tests meet the following conditions: (1) Absolute neutrophil count (ANC) ≥1.5x10^9/L (No G-CSF treatment within 1 week prior to the laboratory test); (2) Platelet count ≥ 75x10^9/L (No platelet transfusion within 1 week prior to the laboratory test); (3) Total bilirubin ≤1.5upper limit of normal (ULN); (4) Aspartate aminotransferase (AST) and alanine aminotransferase (ALT) ≤ 2.5 ULN; (5) Creatinine clearance(Ccr) ≥30mL/min.
7. Females of childbearing potential must have a negative serum beta human chorionic gonadotrophin (β-hCG) pregnancy test result prior to enrollment and must agree to use an effective contraception method for the duration of the study treatment and 7 months after the last dose of study therapy.
8. Males patients and their partners must agree to use an effective contraceptive method for the duration of the study treatment and 4 months after the last dose of study therapy.

The exclusion criteria were:

1. Patients with amyloidosis or central nervous system invasion or on dialysis treatment.
2. Life expectancy < 3 months.
3. History of allergy to mitoxantrone hydrochloride or liposomes; or previous treatment with adriamycin or other anthracyclines, with the total cumulative dose (doxorubicin equivalent) ≥350 mg/m^2.
4. History of allergy (except local injection reaction) or intolerance to bortezomib; or one of the following conditions occurred with prior bortezomib regimens: no treatment response (not reach MR), disease progression within 6 months after the end of last dose.
5. History of contraindications or intolerance to dexamethasone.
6. Any anti-myeloma drug treatment or radiotherapy within 4 weeks prior to the first dose; or enrolled in any other clinical trials of anti-myeloma drug within 3 months prior to the first dose.
7. History of autologous hematopoietic stem cell transplantation within 6 months prior to screening.
8. History of allogeneic hematopoietic stem cell transplantation or solid organ transplantation.
9. Adverse events from the previous treatment have not resolved to ≤ Grade 1 (except for alopecia, hyperpigmentation).
10. Patients with persistent Grade≥2 peripheral neuropathy or Grade 1 peripheral neuropathy with pain.
11. Patients with impaired cardiac function or significant cardiac disease, including but not limited to: (1) Myocardial infarction, viral myocarditis within 6 months before first dose; (2) Heart disease requiring drug treatment before first dose, such as: unstable angina pectoris, chronic congestive heart failure (New York Heart Association (NYHA)≥grade 2), uncontrolled arrhythmias, valvular or persistent cardiomyopathy; (3) Long QTc syndrome or QTc interval > 480 ms; (4) The cardiac ejection fraction is less than 50% in Echocardiography;
12. HBsAg/HBcAb positive with HBV-DNA titer higher than the lower limit of the test value of the research center, or HCV antibody positive with HCV-RNA titer higher than the lower limit of the test value of the research center,or human immunodeficiency virus (HIV) antibody positive.
13. Patients with obvious digestive system dysfunction, which may affect intake, transport and absorption of the study drug.
14. Active bacterial, fungal or viral infections that require systemic treatment within 1 week prior to the first dose.
15. Patients underwent major surgery within 6 weeks prior to the first dose, or had a surgical schedule during the study period;
16. History of additional malignant tumor within 5 years, except for locally curable cancer that has been cured.
17. Other medical conditions that, in the judgment of the investigator, may affect the patient's participation in this study.
18. Pregnant or breastfeeding women;
19. Not suitable for this study as decided by the investigator due to other reasons.

**Table S1. First-line treatment regimens of the 20 patients with RRMM.**

|  | Cohort A  (N=6) | Cohort B  (N=7) | Cohort C  (N=7) | Overall  (N=20) |
| --- | --- | --- | --- | --- |
| First-line treatment，n (%) |  |  |  |  |
| PI+IMiD | 1 (16.7) | 1 (14.3) | 1 (14.3) | 3 (15.0) |
| Bortezomib | 4 (66.7) | 5 (71.4) | 4 (57.1) | 13 (65.0) |
| Lenalidomide | 0 | 1 (14.3) | 1 (14.3) | 2 (10.0) |
| Thalidomide | 3 (50.0) | 2 (28.6) | 0 | 5 (25.0) |
| Bortezomib+Lenalidomide | 0 | 1 (14.3) | 1 (14.3) | 2 (10.0) |
| Steroids | 6 (100) | 6 (85.7) | 7 (100) | 19 (95.0) |
| Chemotherapy | 6 (100) | 5 (71.4) | 5 (71.4) | 16 (80.0) |

PI + IMiD, proteasome Inhibitor (including bortezomib, carfilzomib, ixazomib, etc) + immunomodulatory drug (including lenalidomide, thalidomide, pomalidomide, etc); Steroids, corticosteroids including dexamethasone, prednisone, and their phosphate formulations; Chemotherapy, (including cisplatin, doxorubicin, pegylated liposomal doxorubicin, epirubicin, pirarubicin, cyclophosphamide, etoposide, vincristine, bendamustine, decitabine, iritotecan, etc.).
